# Therapeutic effects of shaogan fuzi decoction in rheumatoid arthritis: Network pharmacology and experimental validation

**DOI:** 10.3389/fphar.2022.967164

**Published:** 2022-08-17

**Authors:** Lu Shi, Yiying Zhao, Chenran Feng, Feng Miao, Linlin Dong, Tianquan Wang, Antony Stalin, Jingyuan Zhang, Jingru Tu, Kexin Liu, Wenyan Sun, Jiarui Wu

**Affiliations:** ^1^ Department of Pharmacology of Chinese Materia Medica, School of Chinese Materia Medica, Beijing University of Chinese Medicine, Beijing, China; ^2^ Institute of Fundamental and Frontier Sciences, University of Electronic Science and Technology of China, Chengdu, China; ^3^ Department of Clinical Chinese Pharmacy, School of Chinese Materia Medica, Beijing University of Chinese Medicine, Beijing, China

**Keywords:** shaogan fuzi Decoction, rheumatoid arthritis, network pharmacology, TLR4/MAPKs/NF-κB signaling pathway, inflammatory

## Abstract

Shaogan Fuzi Decoction (SGFD), one of the classical prescriptions of Chinese Medicine, has a long history in the treatment of rheumatoid arthritis (RA), but definitive studies on its efficacy and mechanism of action are lacking. This study aims to elucidate the pharmacodynamic role of SGFD against RA and the potential mechanisms based on a combination of network pharmacology and experimental verification. The RA model in rats was induced by intradermal injection of bovine type Ⅱ collagen and incomplete Freund’s adjuvant at the tail root. SGFD was administered once a day by oral gavage for 4 weeks. After SGFD administration, rat’s arthritis index (AI) score and paw swelling decreased to some extent, and synovial inflammation, vascular hyperplasia, and cartilage destruction of the ankle joint were improved. Simultaneously, thymus and spleen index and serum levels of C-reactive protein (CRP) were lowered. Network pharmacology revealed that quercetin, kaempferol, naringenin, formononetin isorhamnetin and licochalcone A were the potentialiy active components, and IL6, TP53, TNF, PTGS2, MAPK3 and IL-1β were potential key targets for SGFD in the treatment of RA. Ingredients-targets molecular docking showed that the components had the high binding activity to these target proteins. The mechanism of SGFD for RA involves various biological functions and is closely correlated with TNF signaling pathway, Osteoclast differentiation, T cell receptor signaling pathway, mitogen-activated protein kinase (MAPK) signaling pathway, NF-κB signaling pathway, toll-like receptor signaling pathway, and so on. Western blot and ELISA showed that the expression of toll-like receptor 4 (TLR4), nuclear factor kappa-B (NF-κB) p65, phosphorylated c-Jun N-terminal kinase (p-JNK), p-p38, phosphorylated extracellular regulated kinase (p-ERK) and TNF-α was significantly upregulated in the synovium of RA rats, and the levels of serum inflammatory factors were significantly increased. SGFD inhibits the activation of the TLR4/NF-κB/MAPK pathway and the expression/production of pro-inflammatory cytokines. In summary, SGFD could improve the symptoms and inflammatory response in collagen-induced arthritis (CIA) rat model. The mechanism might be related to the regulation of TLR4/MAPKs/NF-κB signaling pathway and the reduction of inflammatory factor release, which partially confirms the results predicted by network pharmacology.

## Introduction

As a chronic progressive autoimmune disease, the major pathological features of rheumatoid arthritis (RA) are infiltration of inflammatory cells, the aberrant proliferation of synovial cells, formation of pannus, erosion of cartilage and bone, and ultimately leading to destruction, deformation and loss of function of the affected joints. It is associated with a high prevalence of concomitant diseases such as cardiovascular disease, lung disease and other extra-articular and systemic diseases (Mcinnes and Schett, 2011). RA affects 0.5–1% of the global population and represents a significant economic and social burden due to high rates of morbidity, disability, and mortality (Cross et al., 2014). The pathogenesis and aetiology of RA remain unclear; however, immunological, genetic, infectious, environmental, and hormonal factors have been shown to be involved in its development (Guo et al., 2018). Currently, nonsteroidal anti-inflammatory drugs (NSAIDs), disease-modifying antirheumatic drugs (DMARDs), glucocorticoids, and biologics are commonly used in clinics as regular and primary candidates for the treatment of RA (Smolen and Aletaha, 2015; Aletaha and Josef, 2018). Although these agents play a role in relieving symptoms and delaying relapses, they have side effects such as gastrointestinal abnormalities, liver and kidney damage, and bone marrow toxicity (Wang et al., 2018). So it is necessary to explore new therapeutic agents with low toxicity and high efficacy.

According to the theory of traditional Chinese medicine (TCM), RA is classified as a “Bi syndrome” associated with an insufficiency of the body’s vital energy (known as qi), and the intrusion of “wind (feng)”, “cold (han)”, “dampness (shi)”, “heat (re)” and other evil qi from outside, resulting in a blockage of qi and blood in the meridians (Lü et al., 2015; Li and Zhang, 2020). Chinese Medicine has accumulated abundant clinical experience and effective formulas for treating RA. Shaogan Fuzi Decoction (SGFD) was first described in the Treatise on Febrile Diseases (*Shang Han Lun*). It is consists of Paeoniae Radix Alba (*Paeonia lactiflora* Pall., Bai-Shao), Glycyrrhizae Radix et Rhizoma (*G. uralensis* Fisch., *G. inflata* Bat., or *G. glabra* L., Gan-Cao), and Aconiti Lateralis Radix Praeparata (*Aconitum carmichaelii* Debx., Fu-Zi) with the functions of reinforcing deficiency and reducing excess, warming and activating meridians, nourishing blood and extending sinew, relieving spasm and pain (Zhang et al., 2015). In the clinical treatment of RA, SGFD and its modification have shown good efficacy and safety. However, the efficacy and mechanism of action of SGFD have not yet been systematically studied.

**FIGURE 1:**
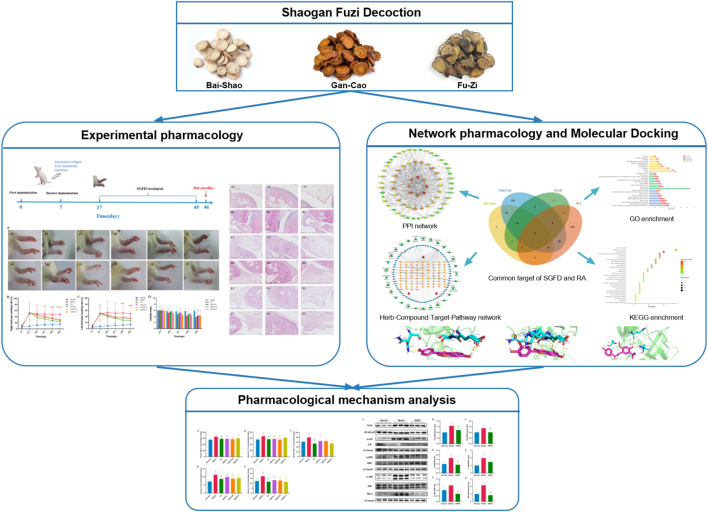
The flowchart of this study based on an integration strategy of network pharmacology and experimental verification.

Network pharmacology is a strategy and technology of bioinformatics network construction and network topology analysis based on a network database query, high-throughput histological data analysis, and computer virtual computing (Hopkins, 2007). It adopts a holistic perspective to systematically elucidate drug mechanisms of action by revealing the relationships between drugs, targets, and diseases. Network pharmacology is consistent with TCM’s multi-component and multi-target features and the ideas of dialectical theory and systematic regulation. It provides a novel idea for studying herbal compounds and has been extensively used to predict the possible active ingredients and targets of TCM and explore its mechanism of action (Liu and Sun, 2012; Yuan et al., 2017). In this study, we used a network pharmacology approach to predict the molecular targets and signaling pathways of SGFD at RA, validate its pharmacological efficacy using the rat model of collagen-induced arthritis (CIA), and preliminarily explore the underlying mechanism. The system flowchart of this work is shown in Figure 1.

## Methods

### Establishment of the collagen-induced arthritis model and drug administration

Wistar rats (SPF grade, male, 170–200 g) were purchased from Beijing Weitong Lihua Experimental Animal Technology Co., Ltd. The license number for experimental animal production is SCXK (Jing) 2016–0011. All rats were housed in a barrier-free environment at the Beijing University of Chinese Medicine, and the license number for experimental animal use is SYXK (Jing) 2020–0033. The animals were housed in a temperature of 22–24°C, relative humidity of 50–70%, and a light-dark cycle of 12 h. All rats were acclimatized to the environment for 1 week before the experiment. The animal study was reviewed and approved by the Medical and Experimental Animal Ethics Committee of the Beijing University of Chinese Medicine (No. BUCM-4-2019100501-4122).

**FIGURE 2:**
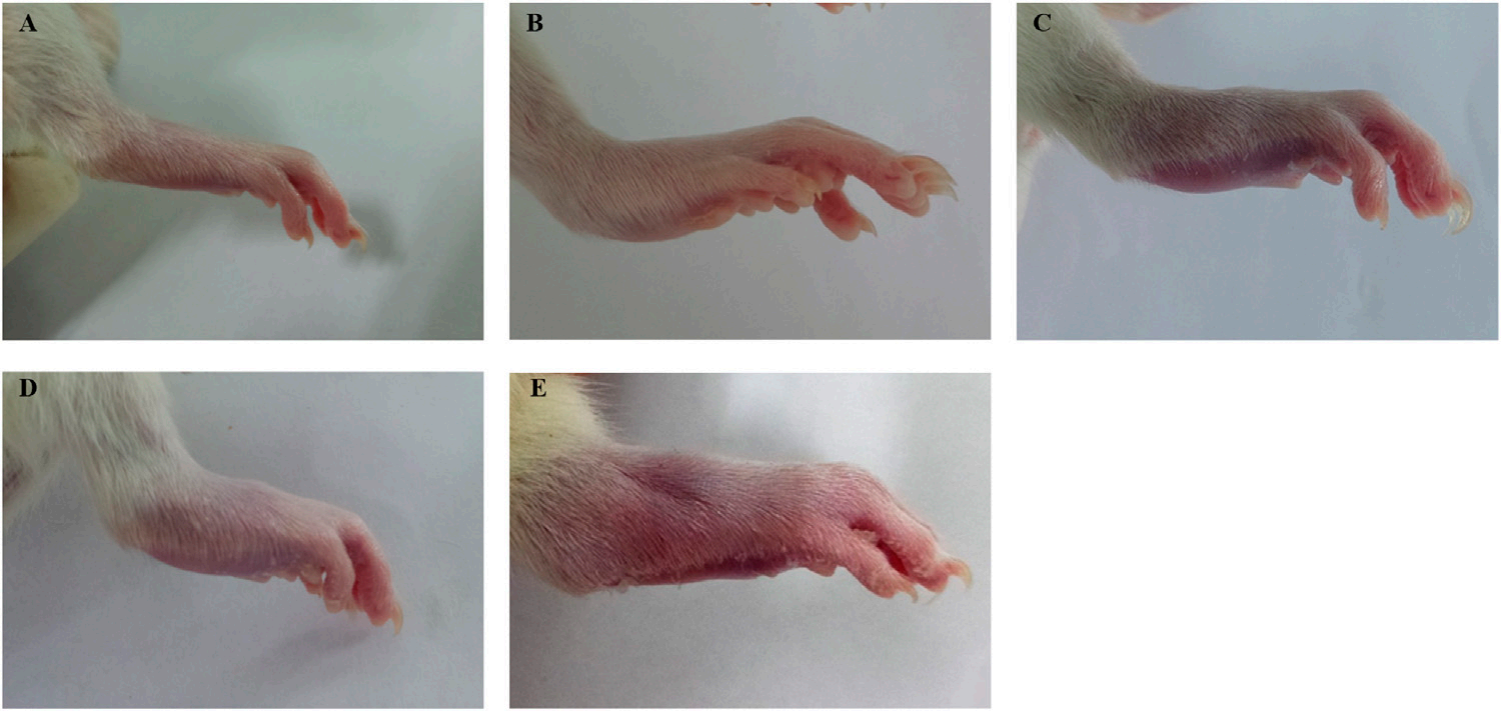
Example of arthritis index score in rats. **(A)** score 0. **(B)** score 1. **(C)** score 2. **(D)** score 3. **(E)** score 4.

Bovine type II collagen (2 mg/ml in 0.05 M acetic acid; Chondrex, Inc., Redmond, WA, United States) was emulsified in an equal volume of incomplete Freunds adjuvant (Sigma-Aldrich, St. Louis, MO, United States). Each rat was immunized on day 0 with 0.2 ml of the cold emulsion by multiple intradermal injections on the back and tail base. On day 7, after the first immunization, 0.1 ml of the emulsified collagen II was injected intradermally at the base of the tail for booster immunization (Trentham et al., 1977; Wang et al., 2019). Normal control rats were injected with an equal volume of saline at the same site and time point. The progression of arthritis was monitored daily. At 17 days after the first immunization, the CIA rats were scored by the arthritis index (AI). The AI was graded on a scale of 0–4 (Brand et al., 2007): 0 = no evidence of hyperemia and/or inflammation; 1 = slight  redness and swelling at the toe joints; 2 = moderate swelling of the toe and ankle joints; 3 = swelling and hyperemia in all areas below the ankle; and 4 = all joints, including the ankle, were swollen (Figure 2). The sum of the four paw scores is the AI of each rat, with a maximum score of 16, and rats with an AI score greater than two were selected as successful rats for the CIA model.

A total of 65 CIA model rats were successfully duplicated and randomly divided into five groups as follows (*n* = 13/group): the model group, the positive drug tripterygium glycosides tablet group (9 mg/kg) (TG), the SGFD group with low-dose (1.05 crude drug g/kg) (SGFD-L), medium-dose (2.1 crude drug g/kg) (SGFD-M) and high-dose (4.2 crude drug g/kg) (SGFD-H). Meanwhile, the normal group was established.

The formula of SGFD was purchased from the Pharmacy Department of Dongfang Hospital, Beijing University of Chinese Medicine (Beijing, China). Each packet contains Bai-Shao 9g, Gan-Cao 9g, and Fu-Zi 3g (in crude drug). TG was purchased from Zhejiang Deende Pharmaceutical Co., Ltd. The tablet and Chinese granules were ground and mixed with distilled water before use. Animals were treated with 10 ml/kg by oral gavage, once a day for 4 weeks (D18 to D45). In addition, rats in the normal and model groups received the same amount of distilled water. The rat’s spontaneous activity, mental status, and weight were observed.

### Evaluation of collagen-induced arthritis

The swelling of the hind paws of rats was measured with a paw volume meter (YLS-7B; Jinan Yiyan Science and Technology Development Co., Ltd.) and the AI of each rat was assessed at days 0, 7, 17, 24, 31, 38, and 45 as described previously. The paw swelling rate was calculated as follows:

Paw swelling rate(%)=(paw volume at the time point − initial paw volume)/initial paw volume×100%

### Histopathological examination

At the end of the experiment, the rats were anesthetized and then sacrificed. The tissues of the right ankle joint were collected and fixed with 4% paraformaldehyde for 24 h and decalcified with 10% EDTA. After processing by conventional histological procedures, including dehydration, transparency, wax immersion, and embedding, the paraffin-embedded tissues were cut into 5-μm-thick sections. Histopathological examination (HE) with staining (G1003, Servicebio, China) was used to assess the pathological and morphological changes in the ankle joint.

### Index of spleen and thymus assay

Thymus and spleen were collected after sacrificed rats, washed in normal saline, blotted with filter paper, and weighed. The thymus and spleen index were calculated as follows:

spleen (thymus) index (‰)=the spleen (thymus) mass (g)/the body weight (g)×1000

### ELISA

The blood of rats was collected by the abdominal aortic method, and the serum was separated by centrifugation. The serum levels of rheumatoid factor (RF), C-reactive protein (CRP), interleukin-1β (IL-1β), IL-6, tumor necrosis factor-α (TNF-α), interferon-γ (IFN-γ), and IL-17 were determined using the respective ELISA kits. The specific operation was performed according to the instructions of the kit.

### Network pharmacology analysis

The ingredients of Bai-Shao (BS), Gan-Cao (GC), and Fu-Zi (FZ) in SGFD were obtained from the Traditional Chinese Medicine systems pharmacology (TCMSP: http://tcmspw.com/tcmsp.php) database (Ru et al., 2014). Oral bioavailability (OB) and drug similarity (DL), as essential pharmacokinetic parameters in the ADME process and drug design, can be used to estimate the druggability of ingredients (Wang and Craik, 2016). Therefore, components with OB ≥ 30% and DL ≥ 0.18 were chosen as active ingredients of SGFD. The SMILE numbers of the active ingredients were acquired from the Pubchem database (Sunghwan et al., 2020) (https://pubchem.ncbi.nlm.nih.gov/) and then sequentially imported into the SwissTargetPrediction database (Daina et al., 2019) (http://www.swisstargetprediction.ch/) for target prediction. Targets with a probability ≥0.5 were selected as potential targets for active ingredients. The TCMSP database was also used to obtain the target information for each active ingredient. RA-related targets were obtained from the Drugbank database (Wishart et al., 2017) (https://www.drugbank.ca/), Therapeutic Target Database (Zhou et al., 2021) (http://db.idrblab.net/ttd/), and DisGeNET database (Janet et al., 2020) (https://www.disgenet.org/) by searching the keyword “rheumatoid arthritis”. The gene names and Uniprot ID of all targets were obtained from the Uniprot database (UP Consortium, 2020) (https://www.uniprot.org/), and the species was restricted to “*Homo sapiens*”.

The overlapping targets between active compound targets and RA-related targets were used to construct a protein-protein interaction (PPI) network by STRING database (Damian et al., 2020) (http://string-db.org/). The species studied was “*Homo sapiens*” with a required minimum interaction score of 0.4. The overlapping targets were imported into the DAVID database (Huang et al., 2009; Wei et al., 2009) (https://david.ncifcrf.gov/tools.jsp) to perform a gene ontology (GO) analysis and a Kyoto Encyclopedia of Genes and Genomes (KEGG) pathway enrichment analysis, where the GO analysis consists of three modules: biological process (BP), Molecular Function (MF) and Cellular Component (CC). Then, the PPI network and the herb-compound-target-pathway interaction network were visualized using Cytoscape 3.7.2 software (Shannon et al., 2003).

### Molecular docking

The top six targets from the PPI network, and the main active compounds of SGFD were selected for the molecular docking analysis. The files of active ingredients in Mol2 format were downloaded from the TCMSP database, and the files of the protein targets in PBD format were downloaded from the PDB database (http://www1.rcsb.org/). AutoDock 4.2 was used to further modify the protein by incorporating new polar hydrogen atoms and calculating the Gasteiger charges. Hydrogen atoms, Gasteiger charges, and rotatable bonds were also assigned to the ligands. Molecular docking was then performed using AutoDock 4.2 and visualized using Pymol software.

### Western bolting

Protein expressions of toll-like receptor 4 (TLR4), nuclear factor kappa-B (NF-κB) p65, c-Jun N-terminal kinase (JNK), p-JNK, extracellular regulated kinase (ERK), p-ERK, p38, p-p38, and TNF-α were measured by Western blotting. RIPA Lysis Buffer containing protease and phosphatase inhibitors was used to lysis synovial tissue from knee joints. Protein concentration was determined using a BCA protein detection kit (P0010S, Beyotime, China). The total protein (10 μg) of each sample was added to SDS-PAGE gel, separated by electrophoresis, and then transferred to the PVDF membrane. The PVDF membranes containing the proteins were placed in a closed solution for 1.5 h and subsequently incubated overnight at 4°C with the required primary antibodies, including TLR4 antibody (1:500, ab13556, Abcam, United Kingdom), NF-κB p65 antibody (1:2000, ab16502, Abcam, United Kingdom), JNK antibody (1:5000, 66210-1-Ig, Proteintech, United States), p-JNK antibody (1:2000, 9255, Cell Signaling Technology, United States), ERK antibody (1:5000, 67170-1-Ig, Proteintech, United States), p-ERK antibody (1:2000, 4370, Cell Signaling Technology, United States), p38 antibody (1:1000, 14064-1-AP, Proteintech, United States), p-p38 antibody (1:1000, 4511, Cell Signaling Technology, United States) and TNF-α antibody (1:1000, 17590-1-AP, Proteintech, United States) and β-tubulin antibody (1:2000, 10094-1-AP, Proteintech, United States) overnight at 4°C. After incubation with HRP-conjugated Affinipure Goat Anti-Mouse IgG (H + L) (1:5000, SA00001-1, Proteintech, United States) or HRP-conjugated Affinipure Goat Anti-Rabbit IgG (H + L) (1:5000, SA00001-2, Proteintech, United States) for 1 h, the PVDF membranes were washed with TBST solution and then processed with chemiluminescent reagents for exposure and photography. Western blot bands were quantified using ImageJ 1.52V and their relative expression levels were quantified as the ratio of the corresponding protein to β-tubulin.

### Statistical analysis

Experimental data were statistically analyzed using SPSS 22.0 and GraphPad Prism 8 software and expressed as mean ± standard deviation (SD). One-Way ANOVA was used when each group’s data were normally distributed and the variances were equal. The Fisher’s LSD post-hoc test was used for comparison between groups. When the data were not normally distributed or the variance was not homogeneous, a nonparametric test was used. The difference was statistically significant when *p*-value < 0.05.

## Results

### Shaogan fuzi decoction treatment alleviated collagen-induced arthritis-related symptoms

**FIGURE 3:**
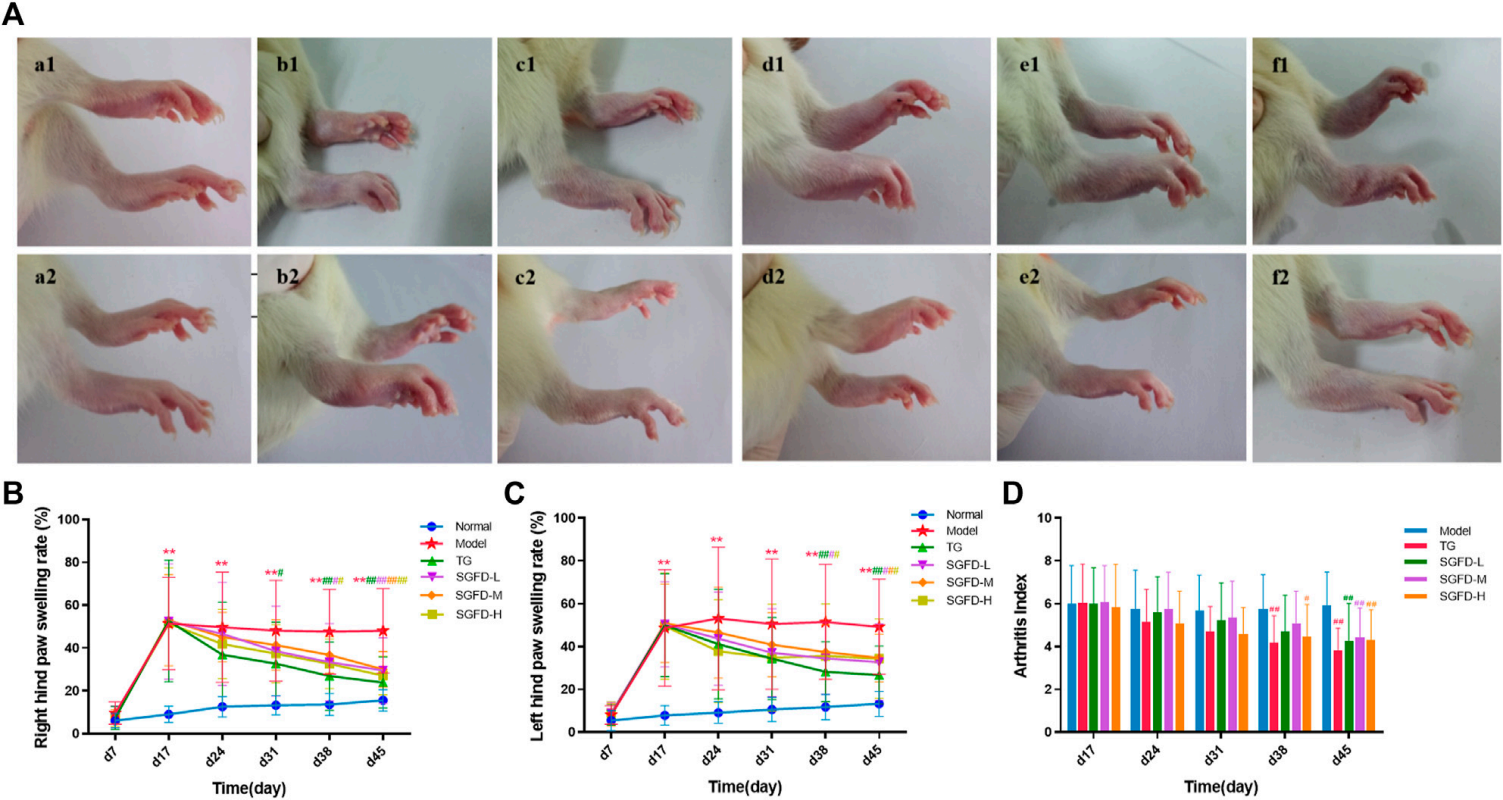
Effect of SGFD on joint swelling in RA rats. **(A)**. Images of rats’ paw swelling in each group (a1-f1: before treatment; a2-f2: after treatment). **(B)**. The results of right hind paw swelling rate. **(C)**. The results of left hind paw swelling rate. **(D)**. The results of AI scores. Data were shown as mean ± SD (Normal, *n* = 13; Model, TG, SGFD-L, SGFD-M, *n* = 12; SGFD-H, *n* = 10). ^*^ *p* < 0.05, ^**^ *p* < 0.01 vs. normal group; ^#^ *p* < 0.05, ^##^ *p* < 0.01 vs. model group.

After booster immunization, redness or swelling of the hind paws and ankle joints was gradually observed in CIA rats. On day 17, 65 rats showed arthritic symptoms, with a success rate of 65%. Moreover, they were randomly divided into five groups and received appropriate treatment. The rats in the normal groups moved freely, showed normal appetite and water consumption and shiny hair, while their weight gradually increased. The rats in the model group exhibited yellowish and lusterless hair, poor appetite, slow weight gain, low mood and activity, and severe paw swelling. After administration of SGFD or TG, the AI score and paw swelling rate decreased compared with the model group. TG and SGFD at high and low doses exhibited the favorable effects of suppressing paw swelling from day 38 (*p* < 0.05 or *p* < 0.01). Similar to the trend of paw swelling results, TG and the high dose of SGFD significantly decreased AI from day 38 (*p* < 0.05 or *p* < 0.01). At the end of the study (day 45), the paw swelling rate and AI of rats in each administration group were significantly lower than those in the model group, and the hair and mental status of rats in the treatment group gradually recovered (Figure 3).

### Shaogan fuzi decoction treatment alleviated the histopathological changes in the joints

**FIGURE 4:**
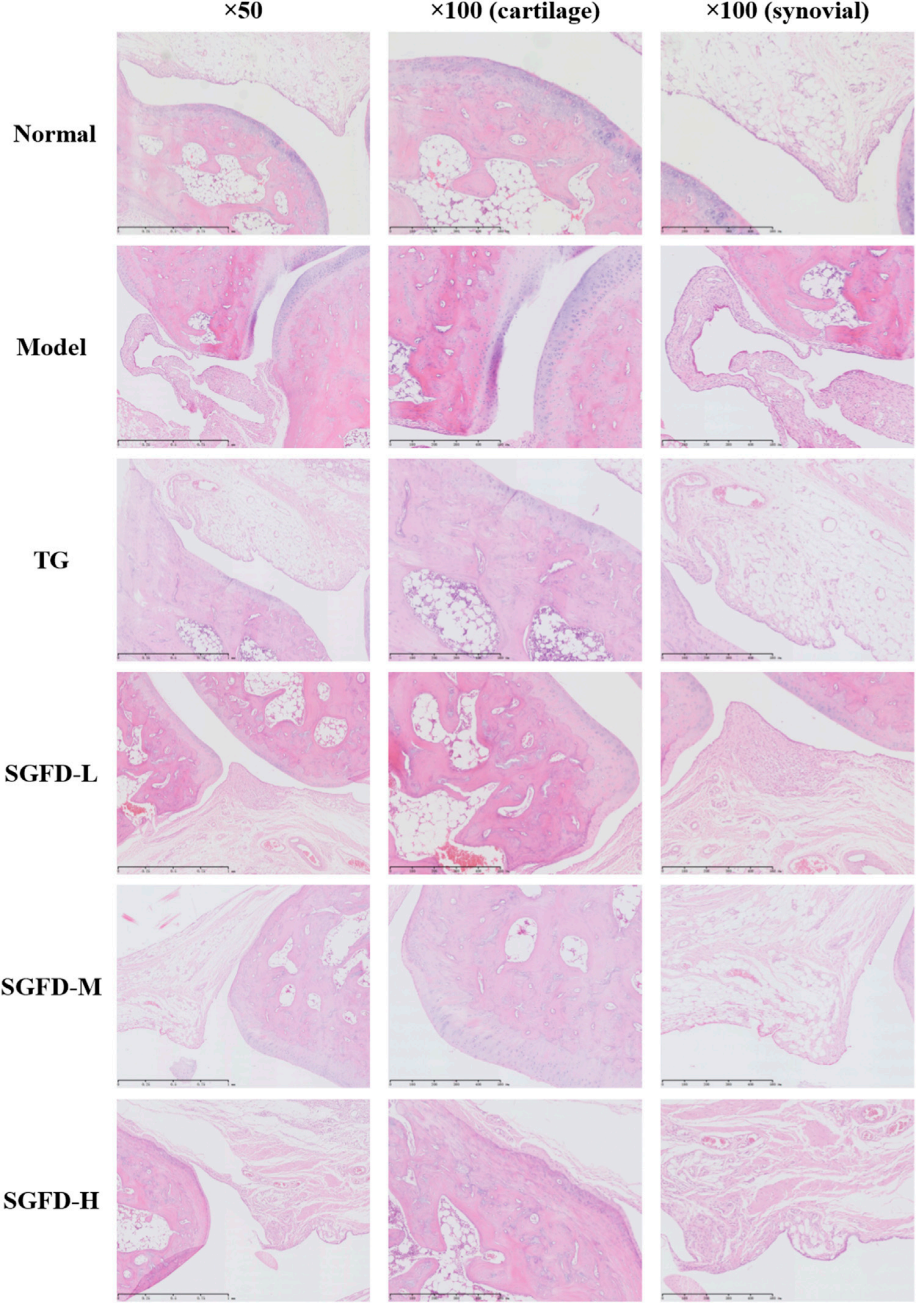
Effect of SGFD on pathological changes of the ankle joint in RA rats.

After treatment, histological analysis of the right ankle joint tissues was performed to further evaluate the therapeutic effect of SGFD. The normal rats showed normal articular cartilage structures with smooth articular surfaces, the chondrocytes were well arranged, with a clear hierarchical structure and uniform distribution, and the nucleus was oval in the center of the cell; the synovial membrane had a regular cellular arrangement, without synoviocyte hyperplasia or inflammatory cell infiltration. In the model group, the articular cartilage surface of the rats was rough, with localized fissure defects, the chondrocytes were disorganized, and the hierarchical structure was blurred; the synovial tissue was hyperplastic and disorganized, accompanied by neovascularization and pannus formation and inflammatory cell infiltration. The degree of ankle joint destruction was significantly improved in all treated rat groups compared with the model group, with mild synovial hyperplasia, destruction of articular cartilage, and less infiltration of inflammatory cells (Figure 4).

### Shaogan fuzi decoction treatment decreased the spleen and thymus index and the levels of rheumatoid factor and C-reactive protein

**FIGURE 5:**
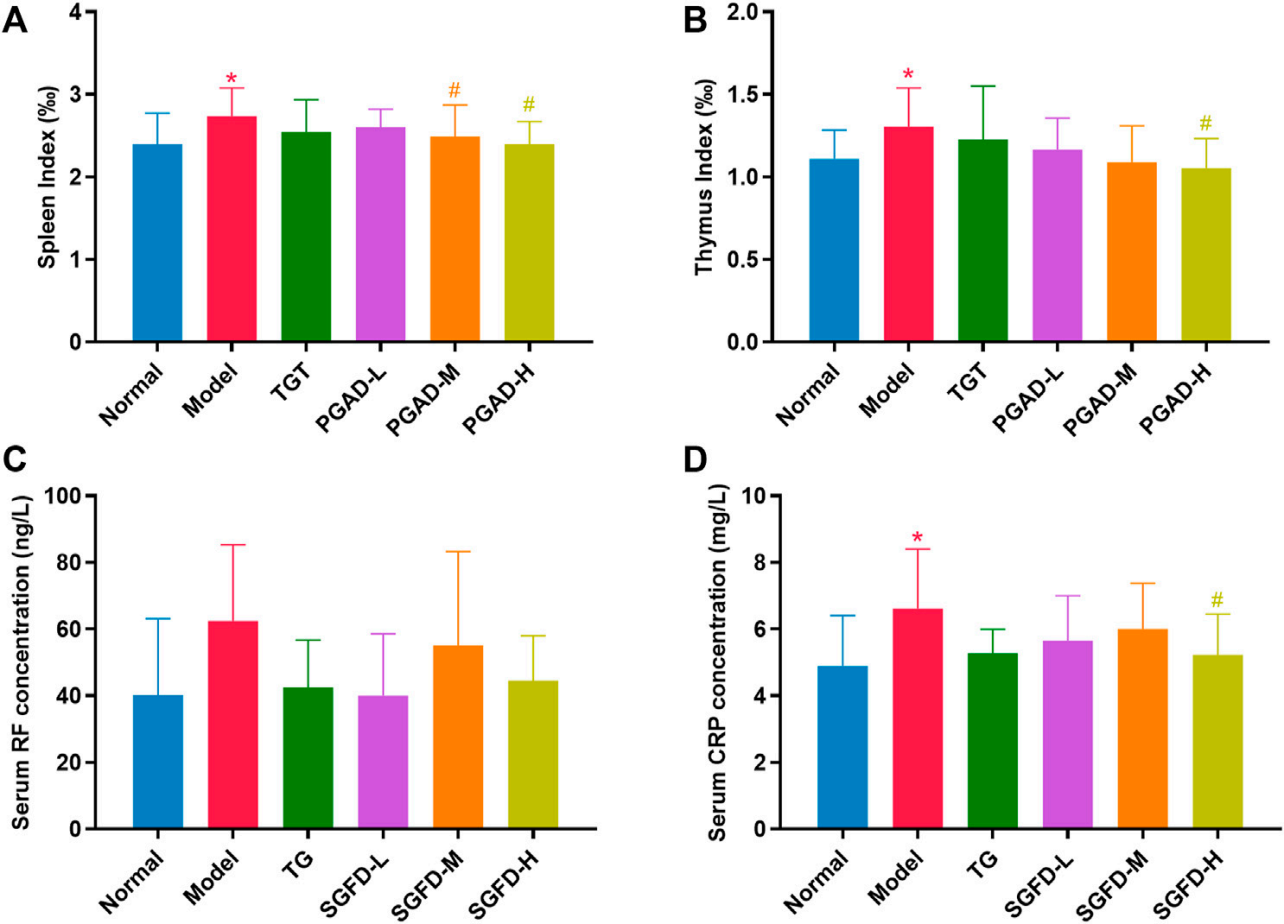
Effects of SGFD on the spleen and thymus index and the serum levels of RF and CRP. **(A)**. Spleen index. **(B)**. Thymus index. **(C)**. Serum RF concentration. **(D)**. Serum CRP concentration. Data were shown as mean ± SD (Normal, *n* = 13; Model, TG, SGFD-L, SGFD-M, *n* = 12; SGFD-H, *n* = 10). ^*^ *p* < 0.05, ^**^ *p* < 0.01 vs. normal group; ^#^ *p* < 0.05, ^##^ *p* < 0.01 vs. model group.

The spleen and thymus index of rats in the model group were significantly increased compared with those in the normal group. Compared with the model group, the spleen index in the SGFD-M and SGFD-H groups and the thymus index in the SGFD-H group showed a significant decrease (*p* < 0.05) (Figures 5A,B). As shown in Figures 5C,D, the serum CRP level was significantly increased in the model group compared with the normal group (*p* < 0.05). Compared with the model group, treatment with SGFD at a high dose significantly decreased the CRP level of rats (*p* < 0.05). Meanwhile, there was no significant difference in RF levels between groups.

### Prediction of targets and pathways by network pharmacology technology

**FIGURE 6:**
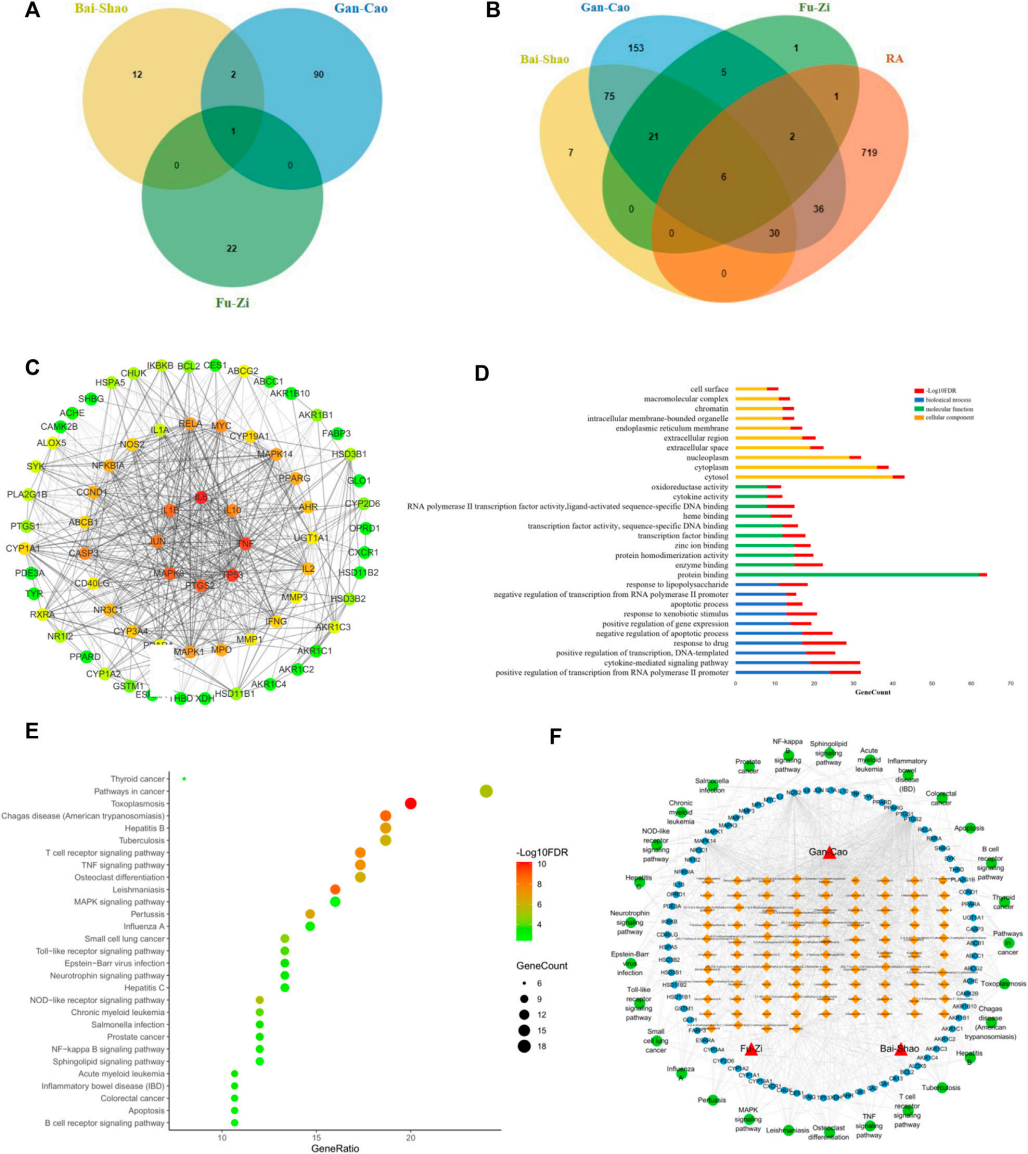
Network pharmacology analysis for screening targets and pathways of SGFD. Venn diagram of **(A)**. Active compounds in SGFD and **(B)**. Common target of SGFD and RA. **(C)**. PPI network of common targets. The target is represented by a circle node. The color of node changed from green to red corresponds to a degree from small to bigger. The thickness of the edge and the combined score value between the protein have a positive correlation. **(D)**. GO enrichment analysis for common targets. **(E)**. KEGG pathway analysis for common targets. **(F)**. Herb-Compound-Target-Pathway network. The red triangles represent the herbal medicines; the yellow squares represent the active chemical compounds of SGFD; the blue dots represent the key targets in the treatment of RA with SGFD; the green dots represent the pathways based on enrichment analysis of key targets.

Firstly, after ADME screening by OB ≥ 30% and DL ≥ 0.18, 127 active ingredients were obtained for SGFD (Figure 6A), and 334 potential human targets corresponded to these compounds. In the corresponding database, 794 targets associated with RA were collected. Through Venn analysis, a total of 75 overlapping targets were screened as key targets for SGFD treatment of RA (Figure 6B). PPI analysis of the above 75 targets was performed using the STRING database, and the network was further constructed using Cytoscape software (Figure 6C). The PPI network consisted of 71 nodes and 619 edges (4 targets were no protein-protein interaction, so they were not shown in the PPI network). There were 30 targets higher than the average degree value, including IL6, TP53, TNF, PTGS2, MAPK3, IL1β, JUN, IL10, CASP3, MYC, RELA, MAPK1, MAPK14, PPARG, MPO (Supplementary Table S1). These proteins might play an essential pharmacological function in the RA process.

To further explore the mechanism of SGFD action on RA at the systemic level, we uploaded 75 overlapping targets to DAVID. GO analysis revealed that these targets were enriched for 114 BP, 10 CC and 52 MF (*p* < 0.01, *FDR* < 0.01). The top 10 terms in BP, CC and MF according to the number of genes are shown in Figure 6D. Enriched BPs included positive regulation of transcription from RNA polymerase II promoter, cytokine-mediated signaling pathway, and positive regulation of transcription. Enriched CCs included cytosol, cytoplasm, and nucleoplasm. Enriched MFs included protein binding, enzyme binding, and protein homodimerization activity. The results of KEGG pathway enrichment analysis showed that 75 key targets were significantly enriched in 29 signaling pathways (*p* < 0.01, *FDR* < 0.01), including TNF signaling pathway, Osteoclast differentiation, T cell receptor signaling pathway, MAPK signaling pathway, NF-κB signaling pathway, Toll-like receptor signaling pathway, etc. (Figure 6E).

An Herb-Compound-Target-Pathway network was constructed to further explore the association between the active ingredients of SGFD and the efficacy of treatment on RA (Figure 6F), consisting of 201 nodes (3 herbal medicines, 94 active chemical compounds, and 75 key targets and 29 pathways). The mean degree value of the active ingredients was 6.8, and 21 compounds had a degree value >7, indicating that most compounds modulate multiple targets to exert different therapeutic effects. Quercetin, kaempferol, naringenin, formononetin, isorhamnetin and licochalcone A, acted on 57, 55, 16, 13, 12 and 11 targets, respectively, indicating that compounds might be crucial for the treatment of RA. The mean degree value of the targets was 11.3, and 20 targets had a degree value >12. The association of multiple targets with multiple compounds suggests that the various components of SGFD act synergistically in the therapeutic process. Moreover, these targets are involved in different pathways and coordinate with each other, suggesting that SGFD may treat RA through multiple pathways and multiple targets. The detailed information is shown in Supplementary Table S2.

### Ingredients-targets molecular docking

**FIGURE 7:**
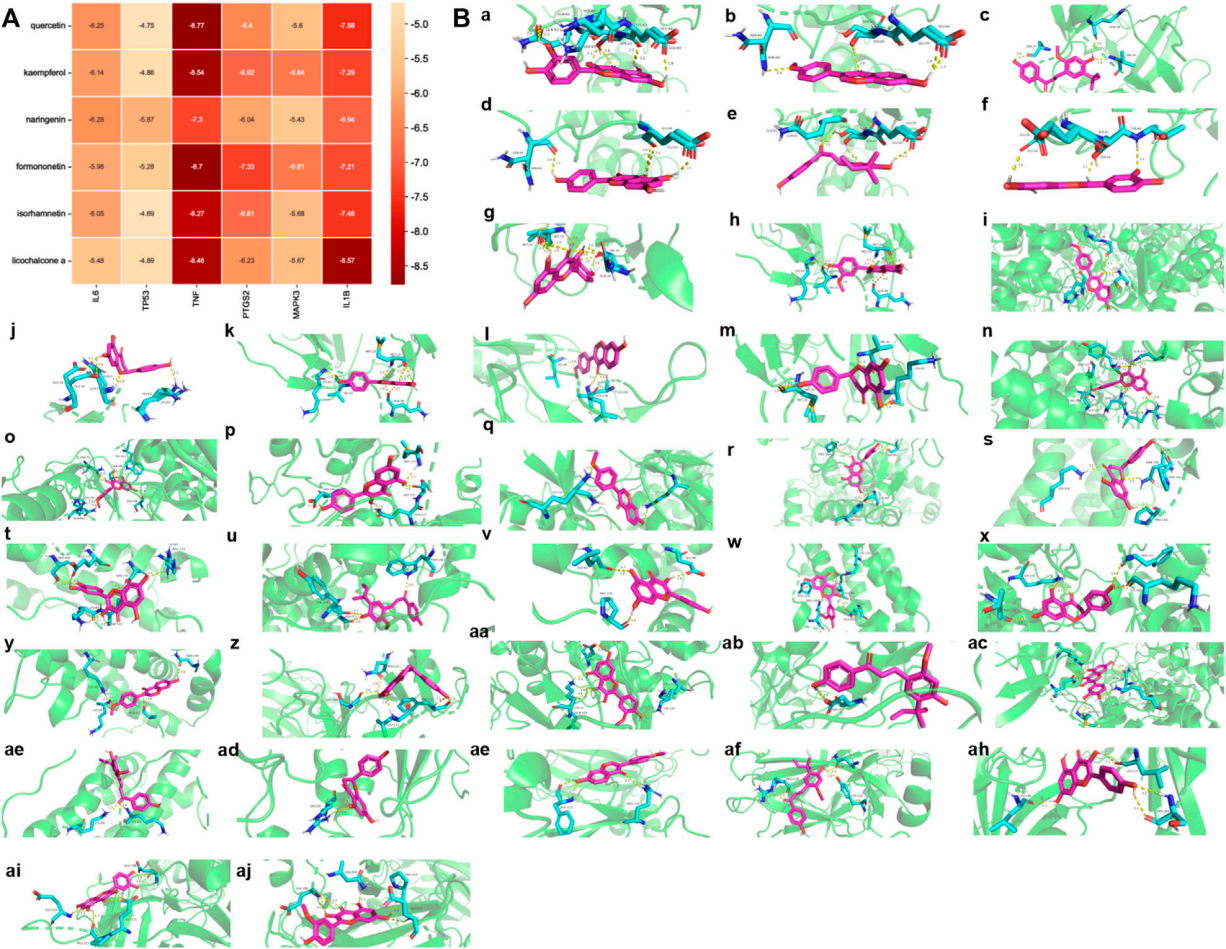
Ingredients-Targets Molecular Docking **(A)**. The binding energy of the main active components of SGFD and the key targets. **(B)**. The binding site of the main active components of SGFD and the key targets. The molecular docking poses of Quercetin—TNF **(a)**, Formononetin—TNF **(b)**, Licochalcone A—IL1β **(c)**, Kaempferol—TNF **(d)**, Licochalcone A—TNF **(e)**, Isorhamnetin—TNF **(f)**, Quercetin—IL1β **(g)**, Isorhamnetin—IL1β **(h)**, Formononetin—PTGS2 **(i)**, Naringenin—TNF **(j)**, Kaempferol—IL1β **(k)**, Formononetin—IL1β **(l)**, Naringenin—IL1β**(m)**, Kaempferol—PTGS2 **(n)**, Isorhamnetin—PTGS2 **(o)**, Kaempferol—MAPK3 **(p)**, Formononetin—MAPK3 **(q)**, Quercetin—PTGS2 **(r)**, Naringenin—IL6 **(s)**, Quercetin—IL6 **(t)**, Licochalcone A—PTGS2 **(u)**, Kaempferol—IL6 **(v)**, Isorhamnetin—IL6 **(w)**, Naringenin—PTGS2 **(x)**, Formononetin—IL6 **(y)**, Naringenin—TP53 **(z)**, Isorhamnetin—MAPK3 **(aa)**, Licochalcone A—MAPK3 **(ab)**, Quercetin—MAPK3 **(ac)**, Licochalcone A—IL6 **(ad)**, Naringenin—MAPK3 **(ae)**, Formononetin—TP53 **(af)**, Licochalcone A—TP53 **(ag)**, Kaempferol—TP53 **(ah)**, Quercetin—TP53 **(ai)**, Isorhamnetin—TP53 **(aj)**.

Molecular docking was used to further evaluate the interaction between components and targets and validate the accuracy of the network analysis. The binding strength of ligand and receptor depends on the binding energy. The lower the binding energy between ligand and receptor, the stronger the stability and the higher the possibility of interaction. The top six core active ingredients quercetin, kaempferol, naringenin, formononetin, isorhamnetin and licochalcone A, and six targets IL6, tumor protein p53 (TP53), TNF, prostaglandin-endoperoxide synthase 2 (PTGS2), MAPK3 and IL-1β were used as ligands and receptors, respectively. Molecular docking results showed that each component had good binding activity to each target (Binding Energy <0 kcal/mol) (Wu et al., 2021) (Figure 7A, Supplementary Table S3). Quercetin, formononetin, kaempferol, isorhamnetin and naringenin showed the best binding activity to TNF with the binding energy of −8.77, −8.70, −8.54, −8.27 and −7.30 kcal/mol, respectively. Licochalcone A showed the best binding activity to IL-1β with a binding energy of −8.57 kcal/mol. The compounds and targets displayed diverse binding patterns to the active sites, including hydrogen bonding, H-π and π-π interactions. Moreover, these compounds bind to the targets through interactions with various amino acid residues, such as LYS-63, GLN-62, GLU-64, ASN-60, SER-65, VAL-41, SER-65, GLN-62. The binding interactions and the binding sites between the compounds and the targets are shown in Figure 7B.

### Shaogan fuzi decoction treatment decreased the levels of inflammatory cytokines

**FIGURE 8:**
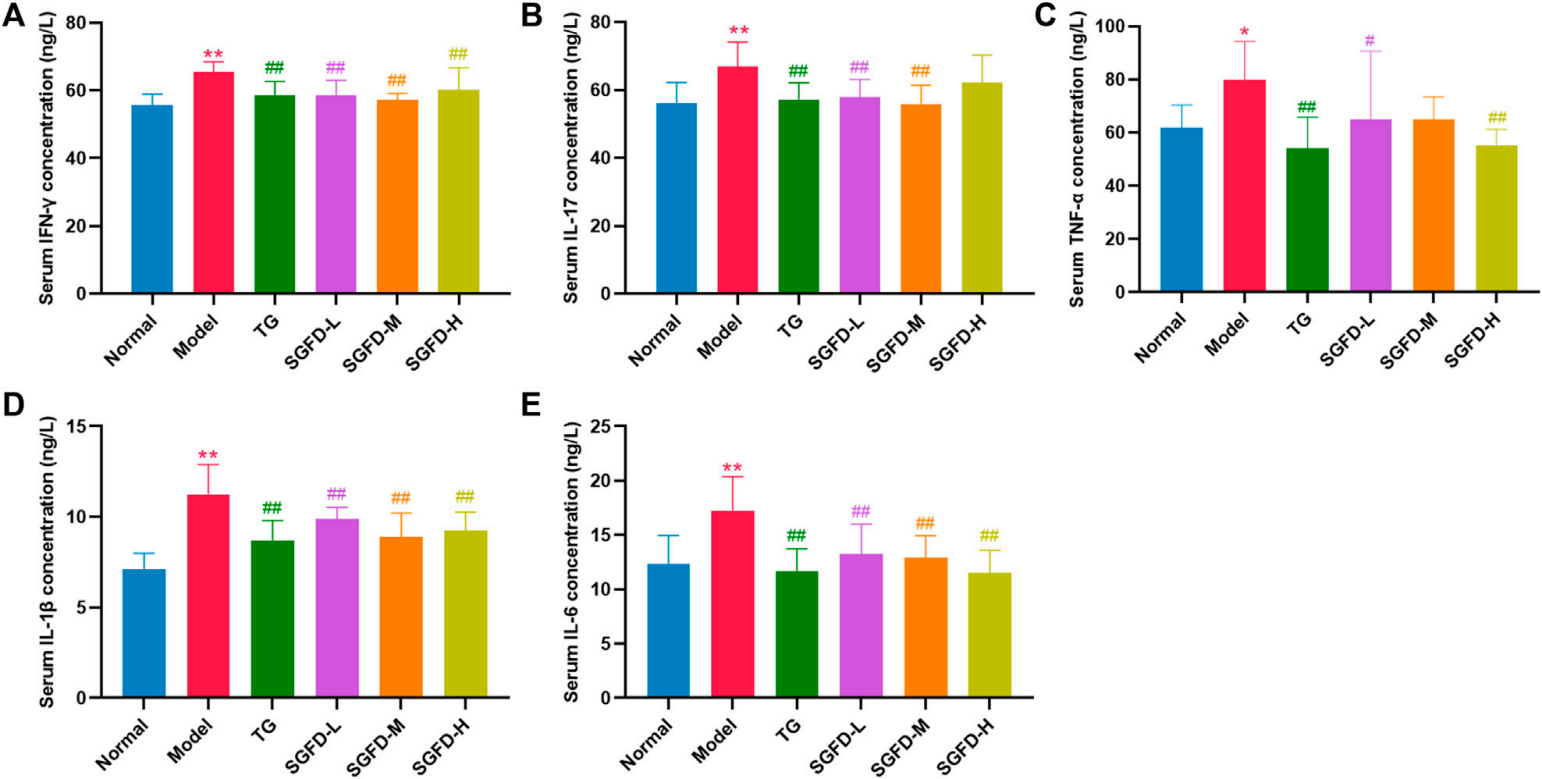
Effects of SGFD on the serum levels of inflammatory cytokines. **(A)**. Serum IFN-γ concentration. **(B)**. Serum IL-17 concentration. **(C)**. Serum TNF-α concentration. **(D)**. Serum IL-1β concentration. **(E)**. Serum IL-6 concentration. Data were shown as mean ± SD (*n* = 8). ^*^ *p* < 0.05, ^**^ *p* < 0.01 vs. normal group; ^#^ *p* < 0.05, ^##^ *p* < 0.01 vs. model group.

As shown in Figures 8A–E, compared with the normal group, serum levels of IFN-γ, IL-17, TNF-α, IL-1β and IL-6 were significantly increased in the model group (*p* < 0.05 or *p* < 0.01). Compared with the model group, SGFD treatment at low and medium doses significantly decreased the levels of IL-17 (*p* < 0.01); SGFD treatment at low and high doses significantly decreased TNF-α level (*p* < 0.05 or *p* < 0.01), and SGFD at all doses notably decreased the serum levels of IFN-γ, IL-1β and IL-6 (*p* < 0.01).

### Shaogan fuzi decoction treatment downregulated the toll-like receptor 4/nuclear factor kappa-B/mitogen-activated protein kinases pathway

**FIGURE 9:**
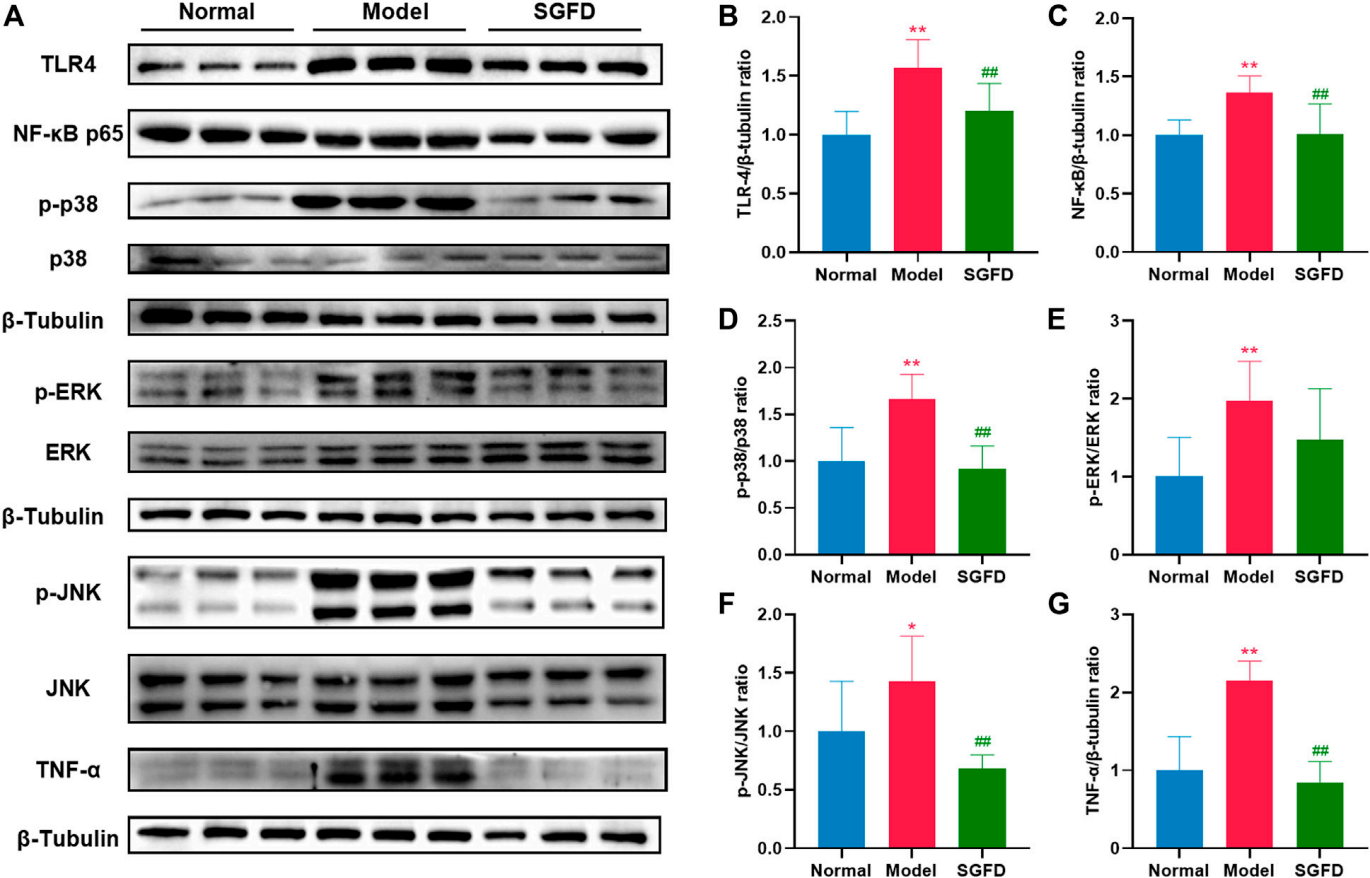
SGFD treatment suppressed TLR4/NF-κB/MAPKs pathway. **(A)**. Western blotting assay was performed to measure the expression of TLR4, NF-κB p65, JNK, p-JNK, ERK, p-ERK, p38, p-p38 and TNF-α in synovial tissues of knee joints **(B–G)**. Relative expression of TLR4, NF-κB p65, p-p38/p38, p-ERK/ERK, p-JNK/JNK and TNF-α were quantified. Data were shown as mean ± SD (*n* = 3). ^*^ *p* < 0.05, ^**^ *p* < 0.01 vs. normal group; ^#^ *p* < 0.05, ^##^ *p* < 0.01 vs. model group.

As shown in Figure 9, protein expressions of TLR4, NF-κB p65, p-JNK, p-ERK, p-p38 and TNF-α and the ratio of p-JNK/JNK, p-ERK/ERK and p-p38/p38 in the synovial tissues of the knee joints were increased in the model groups compared with the normal group (*p* < 0.01). Compared with the model group, a high dose of SGFD treatment decreased the expression of TLR4, NF-κB p65, and TNF-α (*p* < 0.01), and inhibited the increase in phosphorylation of p38 and JNK (*p* < 0.01). The p-ERK1/2 was slightly suppressed but not statistically significant.

## Discussion

RA is a complex, chronic systemic autoimmune disease characterized by joint or synovium inflammation, angiogenesis, cartilage, and bone destruction, leading to joint swelling, pain and morning stiffness, eventually deformity and functional disability (Mcinnes and Schett, 2017). Current RA treatment tends to focus more on improving disease activity. The ultimate goal is to relieve pain and inflammation, maintain joint mobility, and limit the loss of functional capacity (Smolen et al., 2010). SGFD is a representative prescription for the treatment of RA, has a tonic effect on deficiencies, expels wind and dampness, warms the meridians and nourishes the blood to dredge the collaterals, and relieves pain. Clinical studies have shown that treatment with SGFD effectively alleviates clinical symptoms and signs in RA patients and has fewer side effects (Zhang, 2017; Zheng et al., 2018; Zhang, 2021). However, the exact pharmacological effects of SGFD and the major active compounds and molecular mechanisms exerting these effects, have not yet been elucidated. In this study, we demonstrated that SGFD could effectively alleviate the symptoms of RA. The underlying mechanism is related to the suppression of inflammation through the regulation of the TLR4/NF-κB/MAPK signaling pathway.

Currently, the CIA model is the most frequently used animal model to study RA, because it has similar immunological and histological characteristics to human RA patients (Mcnamee et al., 2015; Miyoshi and Liu, 2018). This study successfully established a CIA model in rats, which showed weight loss, joint swelling, pain, inflammation, synovial hyperplasia, and bone and cartilage damage. Tripterygium glycosides tablet is a Chinese patent medicine made from extracts of the traditional Chinese medicinal herb Triptergii Radix (*Tripterygium wilfordii* Hook. f.) (Zhang et al., 2021). It mainly contains diterpenoids, triterpenoids and alkaloids (Zhang et al., 2019). Tripterygium glycosides tablet has anti-inflammatory and suppressive effects on cellular and humoral immunity, and has been approved by the China Food and Drug Administration for the treatment of autoimmune and inflammatory diseases, including RA (State medical license No. Z32021007) (Na et al., 2020). Therefore, tripterygium glycosides tablet was selected as a positive control drug in this study. After treatment with SGFD and tripterygium glycosides tablet, the AI score and paw swelling of rats decreased to a certain extent, and the histopathological lesions in the joints also improved, suggesting that SGFD shows benefit in alleviating the symptoms in RA.

RF is an autoantibody that reacts against the Fc portion of IgG antibodies, and is produced locally by B cells in lymphoid follicles (Moura et al., 2012). The immune complex formed by RF is deposited on the vascular wall and articular cartilage, which can enhance the inflammatory and destructive response (Fang et al., 2019). The titer of RF correlates positively with the severity and active stage of RA. Therefore, RF can be used to determine the severity of RA and the efficacy of treatment (Song and Kang, 2010). In our present study, it was found that the serum RF level slightly decreased in RA rats after SGFD treatment, but it was not statistically significant, which may be due to the lower specificity of RF. CRP is an acute-phase protein, and usually increases rapidly during the acute phase of the host inflammatory response. CRP is a reliable and accurate marker of the inflammatory response *in vivo*, and when combined with RF, can help avoid misdiagnosis (Machold et al., 2007). The serum CRP level was significantly increased in the model group, whereas it was decreased after SGFD treatment. The results of the present study show that SGFD has an effect on reducing RA disease activity.

The pharmacological components and complex molecular mechanism of SGFD on RA were investigated using network pharmacology. By screening the active ingredients and analyzing the Herb-Compound-Target-Pathway network, quercetin, kaempferol, naringenin, formononetin isorhamnetin and licochalcone A were considered as the potential active components of SGFD exerting pharmacological effects on RA. Quercetin (Yuan et al., 2020; Zhao et al., 2020; Costa et al., 2021) has been attributed with a variety of biological activities, such as anti-inflammatory and antioxidant, immunomodulatory, inhibition of MMPs activity and synovial hyperplasia, which can inhibit the pathological process of RA. Kaempferol suppresses proliferation, migration and invasion of fibroblast-like synoviocytes, and alleviates inflammation (Yoon et al., 2013; Lee et al., 2018; Pan et al., 2018). Naringenin (Hajizadeh et al., 2021; Wang et al., 2021), formononetin (Machado Dutra et al., 2021), isorhamnetin (Gong et al., 2020), and licochalcone A (de Freitas et al., 2020; Li et al., 2022) have been reported to have antioxidant, anti-inflammatory, anti-cancer and osteogenic effects, showing great potential as agents for the treatment of RA.

Combined with network pharmacology analysis and related literature, IL6, TP53, TNF, PTGS2, MAPK3 and IL-1β could be potential key targets for SGFD in treating RA. TNF-α, IL-1β and IL-6, as typical pro-inflammatory factors, play important roles in the pathogenesis and development of RA. Currently, TNF-α inhibitory drugs such as the monoclonal antibodies infliximab and adalimumab (Richmond et al., 2015), and IL-6 receptor inhibitors such as the monoclonal antibody tocilizumab, and a monoclonal antibody against IL-1β anakinra are widely used in the clinical treatment of RA (Prieto-Peña and Dasgupta, 2021). PTGS2, also known as cyclooxygenase (COX)-2, is a proinflammatory enzyme that mediates the conversion of arachidonic acid to prostaglandin (PG) E2. This in turn stimulates the production of MMPs, promotes angiogenesis, destruction of cartilage and bone, and inhibits apoptosis of T-cells (Smith et al., 2000). In joint tissue, abnormal expression of the protein COX-2 is an important marker for RA (Kang et al., 1996). A previous study by our group showed that SGFD significantly reduced the expression of PTGS2 protein in the synovial tissues of the ankle of RA rats (Dong et al., 2022). TP53 is a transcription factor that modulates cell cycle initiation. Mutations in the p53 gene (named TP53) may contribute to the tumor-like growth and pro-inflammatory properties of fibroblast-like synoviocytes (FLS), such as aggressive growth of RA-FLS, invasion and destruction of cartilage (Yu et al., 2020). MAPK3, also known as ERK, acts in a signaling cascade that regulates various cellular processes such as proliferation, differentiation and cell cycle progression, and plays an important role in inflammation, proliferation and bone destruction in RA (Ohori, 2008). Molecular docking showed that the active components of SGFD have the high binding activity to these target proteins. This evidence suggests that the pharmacological activity of SGFD against RA is due to the synergistic effect between multiple components and targets.

Among the 29 signaling pathways predicted by network pharmacology, the regulation of inflammatory mediators is an interesting pathway described. RA is a chronic autoinflammatory disease with abnormal innate and adaptive immune responses. The markedly increased thymus and spleen indices in RA rats indicate immune system activation (Steiniger and Meide, 1993). As modulators and effectors of the immune system, cytokines are closely associated with the initiation and development of RA (Hazekawa et al., 2017). The pro-inflammatory cytokines are directly or indirectly involved in the activation and differentiation of pathogenic cells (e.g., Th17), the transcriptional regulation of inflammatory cytokines and chemokines, the processes of angiogenesis, the production of matrix metalloproteinases (MMPs) by synovial cells and chondrocytes, and the development and activation of osteoclasts, leading to synovial inflammation and the destruction of bone and cartilage (Venkatesha et al., 2014). Among the enriched signaling pathways, inflammation-related signaling pathways include the TNF signaling pathway, T cell receptor signaling pathway, MAPK signaling pathway, Toll-like receptor signaling pathway, NOD-like receptor signaling pathway and B cell receptor signaling pathway. T lymphocytes and B lymphocytes are the main cells involved in antigen-specific defense. Activation of the T-cell receptor and B-cell receptor signaling pathways regulates T-cell and B-cell activation, proliferation, differentiation, and death. Abnormal expression of these signaling pathways can lead to defects in the body’s immune response or autoimmune diseases. Pattern recognition receptors (PRRs), represented by TLRs distributed on the membrane surface and Nod-like receptors (NLRs) present in the cytoplasm, play an important role in innate immunity. They recognize pathogen-associated molecular patterns (PAMPs) and damage-associated molecular patterns (DAMPs) and regulates immune responses (Marshak-Rothstein, 2006). Members of NLRs are involved in the control of inflammasome activation, antigen-presentation, signal transduction, transcriptional regulation, and autophagy. NLRs also act as a key regulator of apoptosis and early development. Thus, NLRs are closely associated with various of infection- and immune-related diseases (Kim et al., 2016). TLRs, expressed in a variety of cells such as macrophages, endothelial cells and epithelial cells, are among the most well-studied and well-characterized PRRs. They can detect a variety of PAMPs such as lipids, proteins, lipoproteins and nucleic acids. Activation of TLRs leads to the engagement of diverse intracellular signaling pathways that determine the host inflammatory response. As a member of the toll family, TLR4 activates NF-κB and MAPK signaling pathways through MyD88 dependent or independent pathways and induces the expression of inflammatory cytokines and chemokines (Pålsson-McDermott and O'Neill, 2004). The MAPK signaling pathway can transduce extracellular stimulatory signals into cells to mediate proliferation, differentiation, transformation, apoptosis, and autophagy. MAPK signal transduction pathways associated with RA mainly include the ERK, JNK, and p38 pathways. The abnormally activated MAPK signaling pathway may be involved in the activation, proliferation and differentiation of T cells and induce the expression of inflammatory cytokines, chemokines and MMPs, thereby promoting the inflammatory response of synovitis cells, participating in the excessive proliferation and apoptosis inhibition of synovitis cells, and causing long-lasting chronic synovitis. Moreover, the MAPK signaling pathway induces the expression of MMPs in chondrocytes, regulates chondrocyte apoptosis and osteoclast differentiation, and plays an important role in the process of cartilage and bone destruction in RA joints (Thalhamer et al., 2008). NF-κB is a nuclear transcription activating factor that can initiate or enhance the transcription process of genes and release downstream cytokines such as TNF-α, IL-1β, IL-6, etc (Hayden and Ghosh, 2008). The latter activates NF-κB through a positive feedback loop, resulting in a cascade response that amplifies and sustains inflammation. TLR4, NF-κB and MAPK pathways play important roles in the inflammatory response, and a complex co-regulatory relationship exists between them, that jointly influences the type, magnitude, and duration of the inflammatory response. Given the multi-component, multi-target, and multi-pathway effects of TCM, we used TLR4/NF-κB/MAPK signaling pathway as an entry point to investigate the comprehensive regulatory effect of SGFD on the inflammatory response in RA rats. Our study confirmed that the expression of TLR4, NF-κB p65, p-JNK, p-p38, p-ERK, and TNF-α in the synovium of RA rats was significantly upregulated and suppressed after SGFD administration. Moreover, SGFD treatment significantly reduced the serum levels of TNF-α, IL-1β, IL-6, IFN-γ and IL-17, as well as thymus and spleen indexes in rats, demonstrating that SGFD inhibited the activation of the TLR4/NF-κB/MAPK pathway and the expression/production of pro-inflammatory cytokines at RA.

## Conclusion

**FIGURE 10:**
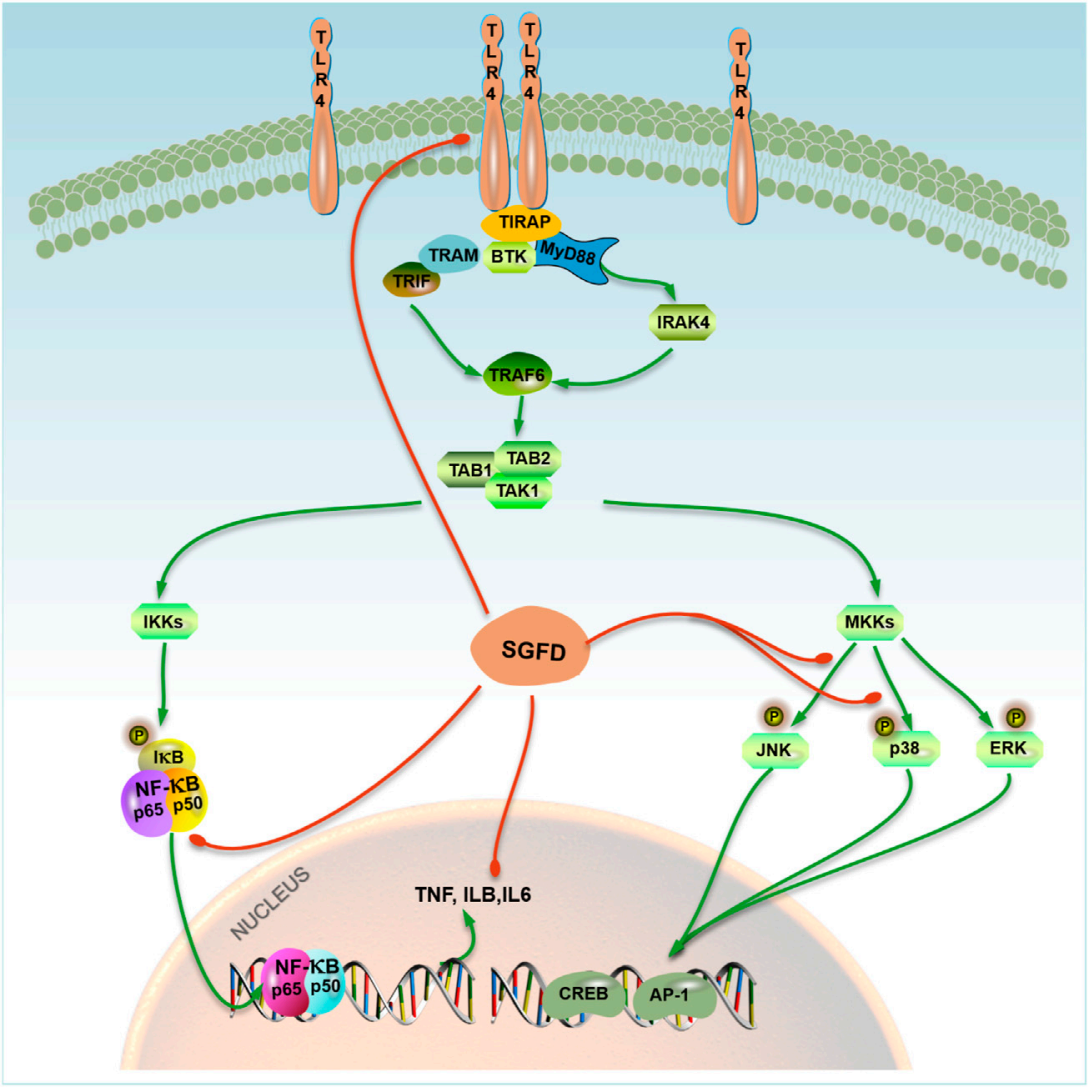
A model representing SGFD molecular targets in TLR4/MAPK/NF-κB pathway. SGFD exerts its anti-inflammatory effect by inhibiting TLR4 and NF-κB expression, the phosphorylation of JNK and p38 in synovial tissues. The action of SGFD is indicated by red lines.

In summary, this study explored the therapeutic effects and underlying mechanism of SGFD against RA based on a combination of network pharmacology and experimental verification. The results showed that SGFD could improve the symptoms and inflammatory response in the CIA rat model. Its mechanism might be related to the regulation of the TLR4/MAPKs/NF-κB signaling pathway, and the reduction of inflammatory factor release (Figure 10), which partially confirms the results predicted by network pharmacology. This study provided the experimental basis for the clinical application and further research of SGFD in treating of RA.

## Data Availability

The original contributions presented in the study are included in the article/Supplementary Materials, further inquiries can be directed to the corresponding authors.
