# Supplementary material for: Therapeutic effects of shaogan fuzi decoction in rheumatoid arthritis: Network pharmacology and experimental validation
Source: Front Pharmacol. 2022 Aug 17;13:967164. doi: 10.3389/fphar.2022.967164 (PMC9428562; doi:10.3389/fphar.2022.967164)
Supplement: Supplementary file 1 [file Table1.docx]

**Supplementary Tables**

**Table S1 Degree of key targets in Treatment of RA with SGFD**

| Degree | Name | Degree | Name |
| --- | --- | --- | --- |
| 45 | IL6 | 27 | NFKBIA |
| 42 | TP53 | 27 | CCND1 |
| 41 | TNF | 26 | NR3C1 |
| 38 | PTGS2 | 26 | IL2 |
| 37 | MAPK3 | 25 | AHR |
| 36 | IL1B | 24 | CYP3A4 |
| 34 | JUN | 23 | NOS2 |
| 32 | IL10 | 23 | IFNG |
| 32 | CASP3 | 22 | CYP1A1 |
| 31 | MYC | 22 | CYP19A1 |
| 30 | RELA | 22 | ABCB1 |
| 30 | MAPK1 | 19 | CD40LG |
| 29 | MAPK14 | 19 | UGT1A1 |
| 28 | PPARG | 19 | MMP1 |
| 28 | MPO | 18 | MMP3 |

**Table S2** **Detailed information on the Herb-Compound-Target-Pathway network**

| Name | Degree | Category |
| --- | --- | --- |
| quercetin | 57 | Compound |
| kaempferol | 55 | Compound |
| naringenin | 16 | Compound |
| formononetin | 13 | Compound |
| isorhamnetin | 12 | Compound |
| licochalcone a | 11 | Compound |
| 7-Methoxy-2-methyl isoflavone | 9 | Compound |
| Glepidotin A | 9 | Compound |
| 7-Acetoxy-2-methylisoflavone | 9 | Compound |
| Calycosin | 8 | Compound |
| Glyasperin C | 8 | Compound |
| (E)-1-(2,4-dihydroxyphenyl)-3-(2,2-dimethylchromen-6-yl)prop-2-en-1-one | 8 | Compound |
| Glypallichalcone | 8 | Compound |
| Licochalcone B | 8 | Compound |
| shinpterocarpin | 8 | Compound |
| Glabrone | 8 | Compound |
| HMO | 8 | Compound |
| 3'-Methoxyglabridin | 8 | Compound |
| 2-[(3R)-8,8-dimethyl-3,4-dihydro-2H-pyrano[6,5-f]chromen-3-yl]-5-methoxyphenol | 8 | Compound |
| Vestitol | 8 | Compound |
| Licoagrocarpin | 8 | Compound |
| Medicarpin | 7 | Compound |
| kanzonols W | 7 | Compound |
| Phaseolinisoflavan | 7 | Compound |
| Glabridin | 7 | Compound |
| Glabranin | 7 | Compound |
| Glabrene | 7 | Compound |
| (2S)-7-hydroxy-2-(4-hydroxyphenyl)-8-(3-methylbut-2-enyl)chroman-4-one | 7 | Compound |
| 1-Methoxyphaseollidin | 7 | Compound |
| Quercetin der. | 7 | Compound |
| Glyasperins M | 7 | Compound |
| Odoratin | 7 | Compound |
| Glycyram | 7 | Compound |
| beta-sitosterol | 7 | Compound |
| PTGS2 | 93 | Target |
| NOS2 | 74 | Target |
| PPARG | 67 | Target |
| MAPK14 | 64 | Target |
| PTGS1 | 46 | Target |
| RELA | 33 | Target |
| RXRA | 28 | Target |
| MAPK1 | 27 | Target |
| ACHE | 26 | Target |
| MAPK3 | 25 | Target |
| JUN | 23 | Target |
| IKBKB | 22 | Target |
| TNF | 22 | Target |
| NFKBIA | 21 | Target |
| CHUK | 20 | Target |
| BCL2 | 18 | Target |
| PDE3A | 15 | Target |
| CASP3 | 14 | Target |
| TP53 | 14 | Target |
| IL6 | 13 | Target |
| Pathways in cancer | 18 | Pathway |
| Toxoplasmosis | 15 | Pathway |
| Chagas disease (American trypanosomiasis) | 14 | Pathway |
| Hepatitis B | 14 | Pathway |
| Tuberculosis | 14 | Pathway |
| T cell receptor signaling pathway | 13 | Pathway |
| TNF signaling pathway | 13 | Pathway |
| Osteoclast differentiation | 13 | Pathway |
| Leishmaniasis | 12 | Pathway |
| MAPK signaling pathway | 12 | Pathway |
| Pertussis | 11 | Pathway |
| Influenza A | 11 | Pathway |

**Table S3** **Molecular docking results of targets and active components**

| Component | Target | PDB ID | Binding Energy(kcal/mol) |
| --- | --- | --- | --- |
| Quercetin | TNF | 6Q00 | -8.77 |
| Formononetin | TNF | 6Q00 | -8.7 |
| Licochalcone A | IL1β | 5R8Q | -8.57 |
| Kaempferol | TNF | 6Q00 | -8.54 |
| Licochalcone A | TNF | 6Q00 | -8.46 |
| Isorhamnetin | TNF | 6Q00 | -8.27 |
| Quercetin | IL1β | 5R8Q | -7.58 |
| Isorhamnetin | IL1β | 5R8Q | -7.46 |
| Formononetin | PTGS2 | 5F19 | -7.33 |
| Naringenin | TNF | 6Q00 | -7.3 |
| Kaempferol | IL1β | 5R8Q | -7.29 |
| Formononetin | IL1β | 5R8Q | -7.21 |
| Naringenin | IL1β | 5R8Q | -6.94 |
| Kaempferol | PTGS2 | 5F19 | -6.92 |
| Isorhamnetin | PTGS2 | 5F19 | -6.81 |
| Kaempferol | MAPK3 | 4QTB | -6.64 |
| Formononetin | MAPK3 | 4QTB | -6.61 |
| Quercetin | PTGS2 | 5F19 | -6.4 |
| Naringenin | IL6 | 1ALU | -6.28 |
| Quercetin | IL6 | 1ALU | -6.25 |
| Licochalcone A | PTGS2 | 5F19 | -6.23 |
| Kaempferol | IL6 | 1ALU | -6.14 |
| Isorhamnetin | IL6 | 1ALU | -6.05 |
| Naringenin | PTGS2 | 5F19 | -6.04 |
| Formononetin | IL6 | 1ALU | -5.98 |
| Naringenin | TP53 | 6FF9 | -5.87 |
| Isorhamnetin | MAPK3 | 4QTB | -5.68 |
| Licochalcone A | MAPK3 | 4QTB | -5.67 |
| Quercetin | MAPK3 | 4QTB | -5.6 |
| Licochalcone A | IL6 | 1ALU | -5.48 |
| Naringenin | MAPK3 | 4QTB | -5.43 |
| Formononetin | TP53 | 6FF9 | -5.28 |
| Licochalcone A | TP53 | 6FF9 | -4.89 |
| Kaempferol | TP53 | 6FF9 | -4.86 |
| Quercetin | TP53 | 6FF9 | -4.75 |
| Isorhamnetin | TP53 | 6FF9 | -4.69 |
